# YOLO11-SBS: leveraging ACE and ORCM to gauge robust and accurate apple counting in complex orchards

**DOI:** 10.3389/fpls.2026.1841916

**Published:** 2026-06-01

**Authors:** Long Zhang, Haochen Wang, Jiarui Zhang, Ye Su, Ruitong Yao, Jiashu Liu, Aowei Jia, Juan Liu, Jie Hu

**Affiliations:** 1 Faculty of Software Technologies, Shanxi Agricultural University, Jinzhong, Shanxi, China; 2 Department of Basic Courses, Shanxi Agricultural University, Jinzhong, Shanxi, China

**Keywords:** causal analysis metric, complex orchard, feature extraction, green apple counting, multi-scale feature fusion, ORCM evaluation metric

## Abstract

Precise apple counting in complex orchards is vital for efficient management, yield estimation, and automated picking. Traditional YOLO-based models suffer from fixed convolutional weights, ineffective feature screening, and interference from branch/leaf shadows and film bagging, leading to poor robustness. They also can’t fully utilize multi-scale feature complementarity, causing detail loss and inaccuracy in counting dense or small apples. To address these, we propose the YOLO11-SBS model. It introduces the C3k2_SAC module for intelligent feature extraction, the C2PSA_BIF module to optimize feature interactions, and adds SSFF and TFE modules to boost feature representation. Furthermore, to comprehensively evaluate model robustness and counting quality, this study applies the ACE metric and constructs a task-oriented Overall Robustness and Counting Metric (ORCM). The ACE metric is used to assess robustness under illumination perturbation, whereas ORCM is designed to jointly characterize false-detection and missed-detection errors under different occlusion levels. The improved model shows marked advantages, reducing missed and false detections. It maintains high accuracy and recall, especially for green apples similar to the background, providing an efficient and accurate solution for apple counting in complex orchards with high practical value.

## 1 Introduction

Apples are among the most widely cultivated fruits worldwide and possess several notable advantages, including strong environmental adaptability, a long storage life, high nutritional value, and stable market demand. As a result, apples occupy a pivotal position in the global fruit industry (Wu et al., 2024). As of December 2025, the apple cultivation area in China reached approximately 1.888 million hectares, with total production projected at 47 million tons, representing a decline of nearly 5% compared with MY 2024/25 (49.3 million tons). China alone accounts for more than 55% of global apple production. Meanwhile, apple imports remain relatively limited at approximately 120,000 tons, while exports are primarily oriented toward neighboring countries (USDA Foreign Agricultural Service, 2025).

Against the backdrop of a continuous decline in the agricultural labor force and the accelerating aging of rural populations, apple harvesting operations are increasingly confronted with severe labor shortages (Liu et al., 2024). One feasible and sustainable solution to this challenge is the introduction of apple harvesting robots into orchard production systems. However, the practical deployment of harvesting robots in real orchard environments faces a series of critical technological bottlenecks closely associated with intelligent decision-making systems, including fruit detection, accurate localization, and path planning (Zhang et al., 2026). Among these challenges, the effective integration of object detection techniques into harvesting robots remains one of the most pressing and fundamental issues. Consequently, apple harvesting robot technology has emerged as a key driver of industrial advancement, with apple detection technology playing a central role in improving harvesting efficiency and operational quality (Liu et al., 2019).

With the continuous advancement and breakthroughs in deep learning technologies, convolutional neural network (CNN)–based object detection models have demonstrated outstanding performance in apple recognition tasks. At present, mainstream object detection approaches can be broadly categorized into two classes.

The first category consists of region proposal–based neural network models, such as R-CNN (Bharati and Pramanik, 2020), Fast R-CNN (Gan et al., 2021), and Faster R-CNN (Cui et al., 2024). Yan et al. (2019)proposed an enhanced Rosa roxburghii recognition method based on Faster R-CNN. In this approach, ROI alignment was adopted, where bilinear interpolation was used to replace ROI pooling in the CNN framework, thereby improving regional feature aggregation. The improved method achieved an average precision of 92.01%. Stein et al. (2016) employed a VGG16-based Faster R-CNN model to detect fruits from multi-view images of mango trees and established correspondences between fruit-counting images using trajectory data provided by a navigation system. Compared with manual counting results, the error rate for a single mango tree was as low as 1.36%.

The second category comprises region-free neural network models (Zhao et al., 2024), including single-shot detection frameworks such as SSD (Nguyen et al., 2025) and YOLO (Deng et al., 2025). Sun et al. (2021) proposed a lightweight MEAN-SSD model for real-time apple leaf disease detection on mobile devices. Experimental results demonstrated that MEAN-SSD achieved a mean average precision (mAP) of 83.12% while maintaining a detection speed of 12.53 frames per second. Yue et al. (2024) designed an ME convolution module to address severe object overlap and branch occlusion issues encountered in the detection of immature citrus fruits in complex environments. This module was integrated into the C2f block of an improved YOLOv8n architecture, based on which a citrus recognition model named YOLOv8-MEIN was proposed. The proposed model achieved an average precision of 96.9%.

Moreover, researchers have extended the application of Transformer models from natural language processing to computer vision tasks (Carion et al., 2020). Compared with convolutional neural networks, Transformers are capable of modeling long-range global dependencies within images and more effectively exploiting contextual information. Although Transformer-based fruit detection methods can capture global image representations, they still suffer from several inherent limitations, including high computational cost, slow convergence, insufficient extraction of local features, and sensitivity to training sample size (Liu et al., 2021). Consequently, these approaches are not well suited for green apple object detection tasks.

Although the above studies demonstrate the effectiveness of mainstream detection frameworks in fruit recognition, research more directly related to apple detection suggests that this task remains highly challenging under real orchard conditions. In recent years, YOLO-based object detection models have achieved notable success in fruit detection applications. (Zhang et al., 2023). innovatively integrated DenseNet into the YOLOv4 network architecture and proposed an AP-Loss function as the training objective. The improved YOLOv4 model exhibited significant improvements in both detection accuracy and inference speed. Kurtulmus et al. (2011) trained and recognized images of green apple trees captured under natural illumination conditions using a combination of Eigenfruit features, color features, and Gabor texture descriptors, achieving a recognition accuracy of 75.3%.

In addition, several studies have employed deep learning–based image detection methods to estimate fruit yield by counting fruits on trees. Koirala et al. (2019) proposed a network termed MangoYOLO for detecting mango fruits across all images collected in orchards and estimating orchard fruit load by aggregating detection counts. However, the resulting estimates exceeded the actual fruit load by 4.6% to 15.2%.(Liu et al., 2024)proposed an MAE-YOLOv8 model based on YOLOv8s-p2 as the baseline to accurately detect green plums in real and complex orchard environments. The proposed model achieved a precision, recall, mean average precision, and detection speed of 92.3%, 82.0%, 89.4%, and 68 frames per second, respectively, representing improvements of 1.4%, 4.0%, 2.9%, and 13.0% over the baseline model.

Despite these advances, several limitations remain insufficiently addressed in existing apple detection studies. First, apple detection is highly task-specific, especially for green apples. In real orchards, green apples often exhibit strong color and texture similarity to surrounding foliage, while branch shadows, fruit bagging, overlapping fruits, and variable illumination further increase the difficulty of reliable feature extraction. Second, many existing studies mainly emphasize detection or segmentation accuracy in single images, but pay limited attention to the direct relationship between detection errors and counting reliability. In practical fruit counting, both false detections and missed detections directly contribute to counting deviation, yet conventional metrics often fail to distinguish their effects under different orchard conditions. Third, although recent studies have extended fruit detection to yield estimation, stable counting under complex orchard disturbances remains insufficiently studied, particularly for dense, small-sized, and partially occluded green apples.

Traditional apple detection metrics, particularly for green apples, often fail to account for several critical real-world complexities. These include lighting variations, background interference, and occlusion caused by leaves or fruit bags, leading to inaccurate counts and reduced model robustness. Existing metrics like precision and recall typically focus on detection accuracy but fail to adequately evaluate the model’s robustness under varied environmental conditions. This limitation is particularly evident in the context of green apple detection, where background clutter and color similarity with surrounding foliage present significant challenges.

Current research on apple recognition has predominantly focused on red apples, whereas green apple detection remains significantly more challenging due to complex background characteristics. In particular, the high similarity between green apples and surrounding foliage in terms of color and texture substantially increases detection difficulty (Zhao and Zhou, 2017). To address this issue, this study applies the Average Causal Effect (ACE) (Zhao et al., 2025) metric and constructs a task-oriented ORCM for occlusion-aware fruit counting evaluation. The ACE metric enables causal-theory-based assessment under illumination perturbation, whereas the ORCM is used to jointly evaluate false-detection and missed-detection errors under different occlusion levels. To address this issue, this study proposes a novel YOLO11-SBS model. Specifically, a C3k2_SAC module is designed to effectively suppress interference caused by branch shadows and plastic fruit bags through dynamic weight calibration and a dual-branch adaptive fusion mechanism. A C2PSA_BIF module is further introduced, which employs window-level feature selection and pixel-level attention enhancement to eliminate irrelevant disturbances such as fruit stems. In addition, SSFF and TFE modules are incorporated to improve counting accuracy for dense and small-sized apples by leveraging a lightweight, parameter-free multi-scale feature fast alignment and aggregation mechanism, along with a 3D convolution–based deep cross-scale feature interaction strategy.

Experimental results demonstrate that the proposed model achieves a well-balanced optimization between recall and precision, with the recall reaching up to 83.7% and precision maintained at 86.7%. The model further attains an mAP@50 of 92.0% and an mAP@50:95 of 75.2%. Moreover, compared with other YOLO baseline models, the proposed approach achieves the best performance in terms of the ORCM metric.

This paper focuses on the image collection of green apple trees during their growth period under natural lighting conditions, aiming to accurately extract the relevant feature information of apple tree fruits in natural lighting environments.

Key Contributions:^4^YOLO11-SBS Model: We propose a novel YOLO11-SBS model that enhances the detection and counting accuracy of green apples in complex orchard environments. This model incorporates advanced modules such as C3k2_SAC, C2PSA_BIF, SSFF, and TFE to optimize feature extraction and interaction.^5^Average Causal Effect (ACE) Metric: We introduce the ACE metric, a novel evaluation approach based on causal theory, which allows for a more accurate assessment of the model’s performance under varying environmental conditions. The ACE metric helps quantify the direct causal impact of phenotypic features (such as shape, size, and spatial arrangement) on the counting accuracy of apples under different lighting scenarios.^6^Overall Robustness and Counting Metric (ORCM): We propose the ORCM, a comprehensive evaluation metric designed to assess the model’s robustness and counting accuracy across multiple scenarios, such as long-distance, short-distance, front-light, back-light, few-fruit, and many-fruit situations. The ORCM allows for a more targeted and scientifically rigorous evaluation of the model’s performance, particularly in complex orchard environments where occlusion and background clutter are prevalent.

The paper is organized as follows: Section 1 provides an Introduction, where we introduce the problem of green apple detection and counting in complex orchard environments, outline the challenges faced by traditional models, and propose the YOLO11-SBS model as a solution. Section 2 presents the Data Collection and Processing methodology, where we detail the datasets used for training and evaluation, describe the annotation process, and explain how the dataset is split into training, validation, and test sets. Section 3 discusses the Model Architecture, providing an in-depth description of the proposed YOLO11-SBS model and the various modules it incorporates, including C3k2_SAC, C2PSA_BIF, SSFF, and TFE, highlighting their individual roles in enhancing detection and counting accuracy for green apples. In Section 4, we introduce and explain the evaluation metrics used in this study, focusing on both traditional metrics as well as the newly proposed Average Causal Effect (ACE) and Overall Robustness and Counting Metric (ORCM), and discuss their application in rigorously assessing the robustness and accuracy of the model. Section 5 presents the Results and Analysis, where we provide experimental results, compare the performance of our model with other state-of-the-art models, perform ablation studies, and offer a detailed analysis using the ACE and ORCM metrics. Finally, Section 6 concludes the paper by summarizing the key contributions, discussing the implications of the findings, and suggesting directions for future work in the field of green apple detection and counting in complex environments.

## 2 Data collection and processing

**Figure 1:**
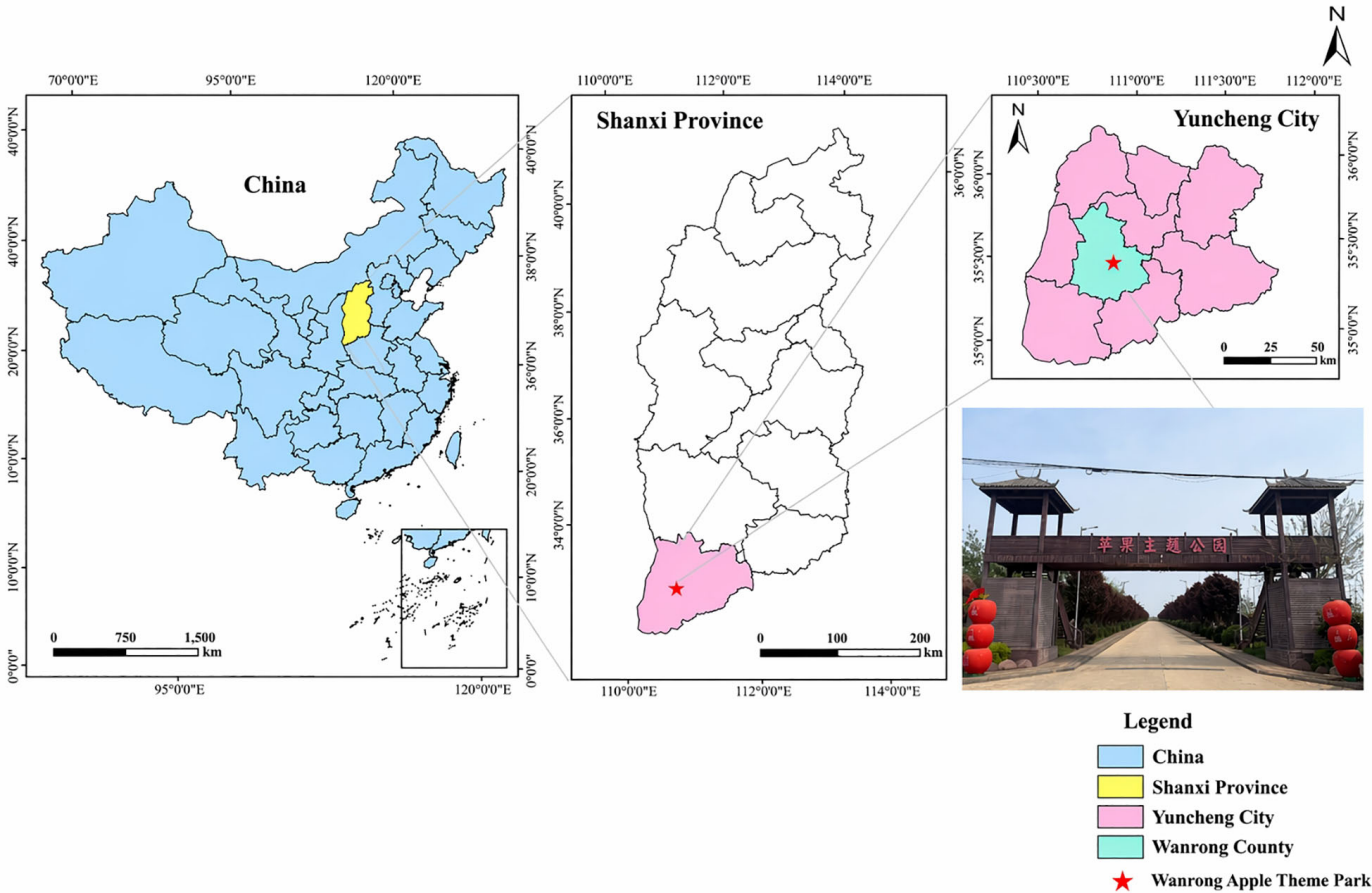
Overview map of the study area.

**Figure 2:**
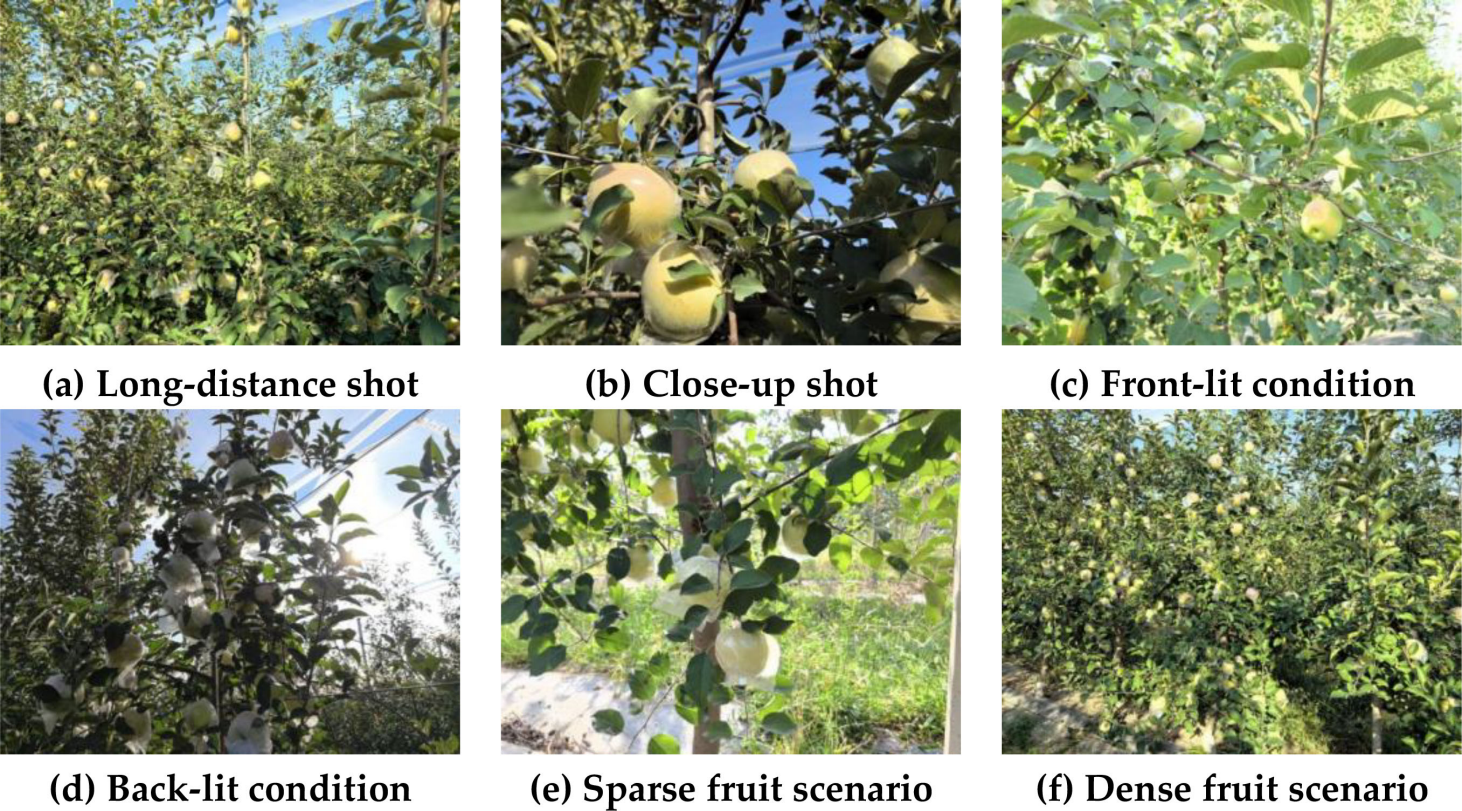
Images of green apples under different orchard scenarios: **(a)** long-distance shot; **(b)** close-up shot; **(c)** front-lit condition; **(d)** back-lit condition; **(e)** sparse fruit scenario; and **(f)** dense fruit scenario.

As shown in **Figure 1**, the data collection site was located in an apple orchard in Wanrong County, Yuncheng City, Shanxi Province, China. The experimental samples were *Ruixue* apples. Image acquisition was conducted from September 20 to October 10, 2025, with data collected at 3-day intervals. To improve dataset diversity and scene representativeness, apple images were captured from different viewing angles and distances, covering a variety of conditions, including multi-fruit scenes, sparse-fruit scenes, front-lighting conditions, backlighting conditions, close-range views, and long-range views. A total of 2,210 images were obtained, with some of them shown in **Figure 2**.

The LabelImg tool was employed to annotate the positional coordinates and categories of apples in the images. This ensured that each annotated rectangular bounding box contained the minimal possible background area, with the category attribute designated as “apple”. The annotations were exported in YOLO-format TXT files, in which each object instance is represented by five values: class ID, normalized (x_center, y_center, width, height). Here, x_center and y_center denote the normalized coordinates of the bounding-box center relative to the image width and height, respectively, while width and height denote the normalized size of the bounding box. Since this study focused on fruit detection and counting rather than disease diagnosis, no disease categories were involved. Subsequently, using Python software, the annotated dataset was randomly divided into training, validation, and test sets in an 8:1:1 ratio, as detailed in **Table 1**. This division ensured that there was no overlap of images across the different sets, thereby preventing overfitting during the model training process.

**Table 1:** Details of the dataset partition for model training and evaluation.

Dataset	Number of images
Training Set	1,768
Validation Set	221
Test Set	221

## 3 Improved YOLO11 network architecture model

**Figure 3:**
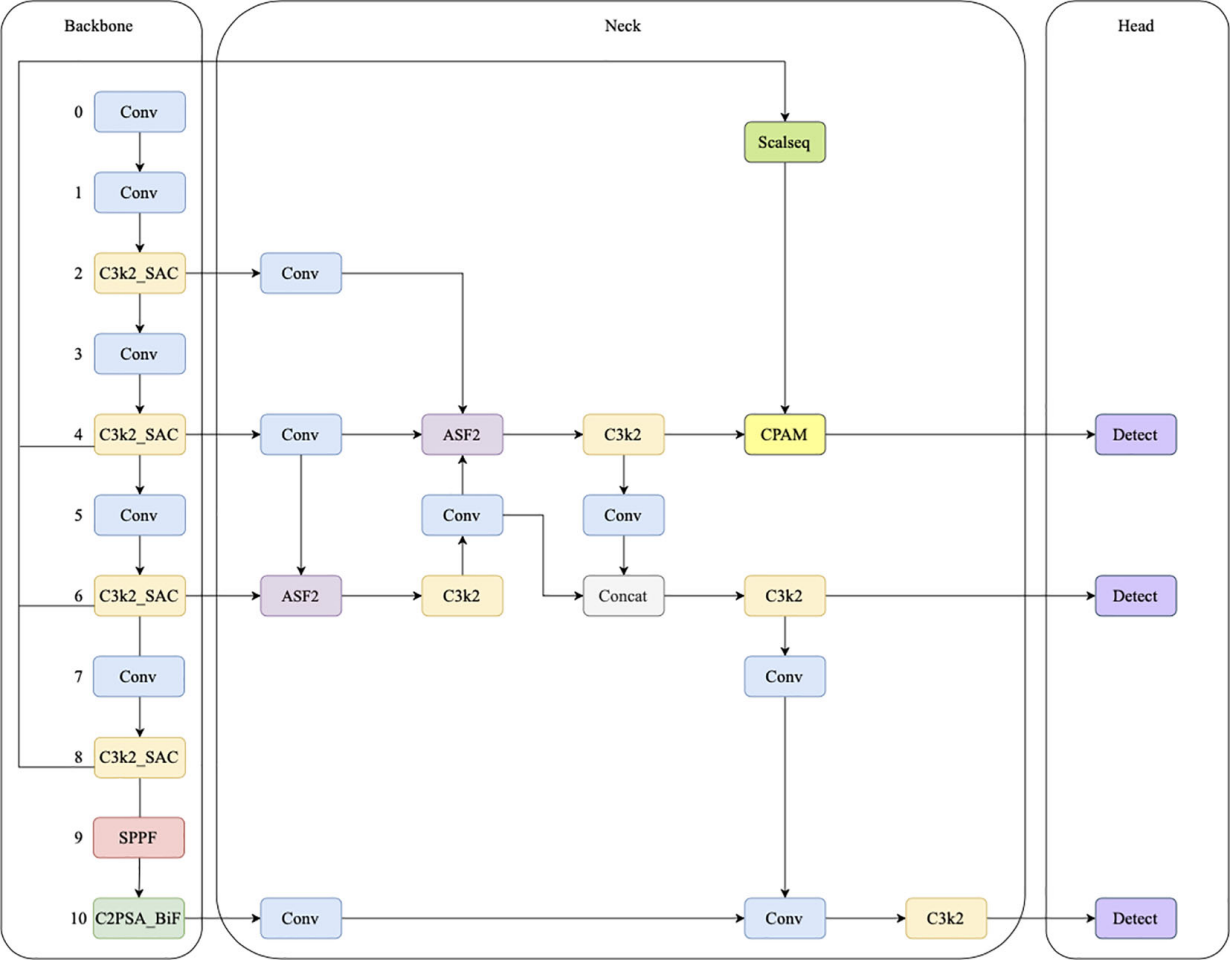
Architecture diagram of the YOLO11-SBS network.

YOLO11 offers the advantages of fast inference speed and lightweight deployment, demonstrating efficient feature extraction and object localization capabilities in general-purpose object detection tasks. This makes it a solid foundational model for the complex task of apple counting in orchards. However, in the intricate scenarios of orchard environments, the original convolutional layer weights of YOLO11 exhibit a fixed distribution, which struggles to adapt to the spatial feature heterogeneity in occluded regions. Additionally, it lacks an effective feature screening mechanism, rendering it susceptible to interference from background elements such as branch and leaf shadows, as well as film bagging. Moreover, its exploitation of the complementary value of multi-scale features remains insufficient, thereby limiting the counting accuracy for densely packed and small-sized apples. To address these challenges, we propose the YOLO11-SBS model, as illustrated in the accompanying **Figure 3**.

The C3k2_SAC module leverages a weight dynamic calibration and a dual-branch adaptive fusion mechanism to overcome the robustness deficiencies caused by the fixed convolutional weights in the original model. Simultaneously, it enables intelligent screening of input features, effectively suppressing interference from branch and leaf shadows, film bagging, and other background elements. The C2PSA_BIF module employs a combination of dual-layer routing attention and position-sensitive attention within the PSABlock, addressing the challenge of traditional attention modules in precisely focusing on critical local features of apples. Through window-level feature screening and pixel-level attention enhancement, it further eliminates interference from irrelevant information such as fruit stems. The proposed ASF2 module, which consists of the SSFF and TFE submodules, employs a lightweight parameter-free scale-aligned aggregation strategy together with a 3D cross-scale feature interaction mechanism. This design enhances the complementary fusion of multi-scale features and improves the counting accuracy of densely distributed and small-sized apples.

### 3.1 C3k2_SAC

**Figure 4:**
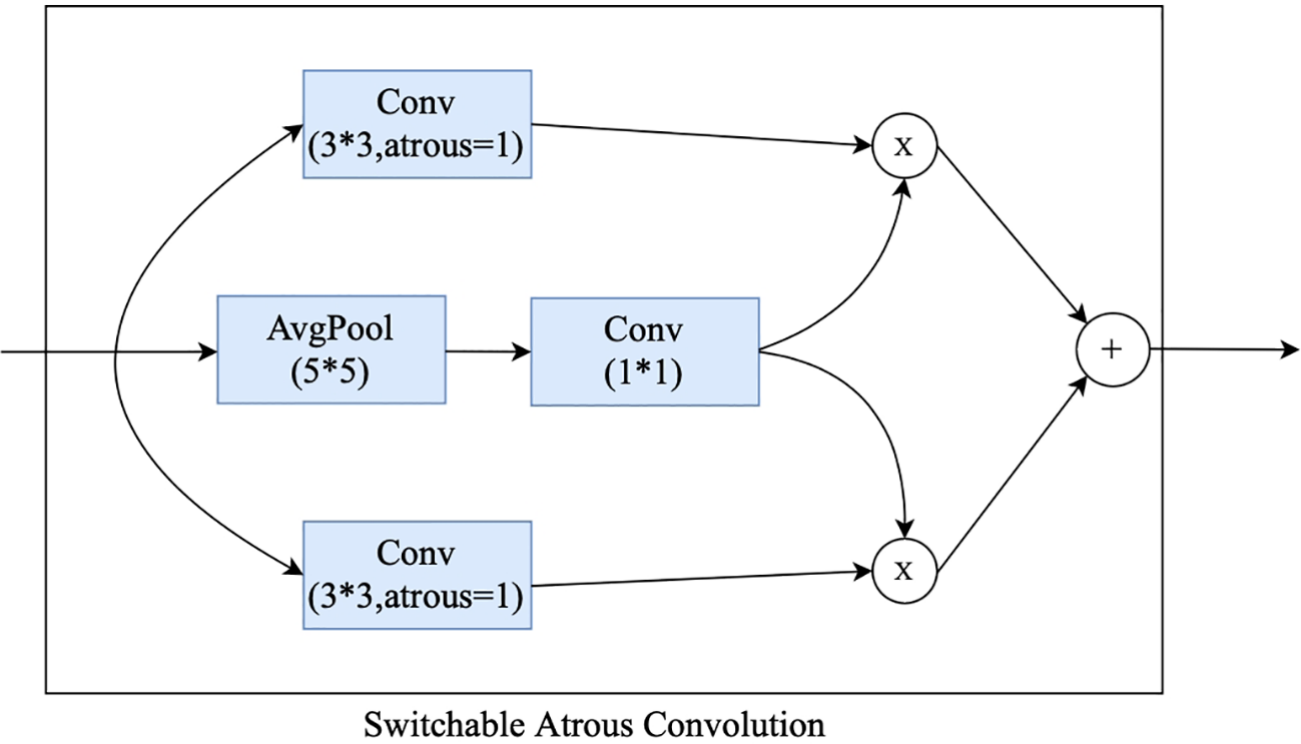
C3k2_SAC module.

In the YOLO model, the weights of the original convolution layer are fixed, which cannot be adaptively adjusted according to the spatial differences of the occluded regions in the input features, resulting in insufficient robustness. In addition, it lacks the capability of feature screening and is susceptible to interference from irrelevant information such as foliage shadows and plastic film bagging. To address these issues, SAConv is introduced and integrated with C3k2 to form a novel module named C3k2_SAC, whose architecture is illustrated in the **Figure 4**. By virtue of dynamic weight calibration and adaptive dual-branch fusion, the module achieves intelligent extraction of input features.

C3k2_SAC is an efficient hybrid CSP feature fusion module improved upon the C2f module, and its overall architecture is divided into three layers with a good balance between accuracy and inference efficiency, as shown in Equation 1. The first layer is the input splitting layer, which adjusts the number of input channels via convolution and then splits the feature map into two parallel branches along the channel dimension, where each branch has the number of channels equal to the dimension of the hidden layer. The second layer is the core module layer, which constructs multiple configurable sub-modules supporting two options: the C3k module and the Bottleneck module embedded with SAConv. These sub-modules process one of the split branches in a serial manner to achieve progressive enhancement of features. The third layer is the feature fusion layer, which concatenates the original split branch with the outputs of all core sub-modules along the channel dimension, and then compresses the number of channels to the target dimension through convolution, thus completing the aggregation and unified output of multi-branch features.

(1) $$Y=\text{Conv}(\text{Concat}(X_{1},M⊙F_{s}+(1-M)⊙F_{l}))$$

Where $X_{1}$ denotes the retained shortcut branch after channel splitting, $F_{s}$ and $F_{l}$ denote the features extracted by the small-receptive-field and large-receptive-field convolution branches, respectively, M denotes the adaptive spatial switching mask, ⊙ denotes element-wise multiplication, and Y denotes the final output feature of the module.

SAConv consists of four core components: first, the weight calibration layer, which realizes dynamic normalization and calibration of convolution weights; second, the switch branch layer, which includes a convolution layer for generating spatial attention masks and a dual-convolution branch with different receptive fields, achieving dynamic fusion of the two branches through weighted calculation; third, the context enhancement layer, which separately extracts and superimposes global context information for both input features and fused branch features to improve feature correlation; fourth, the output activation layer, which is composed of the SiLU activation function, completing batch normalization and nonlinear transformation of features to output the final optimized features.

### 3.2 C2PSA_BIF

**Figure 5:**
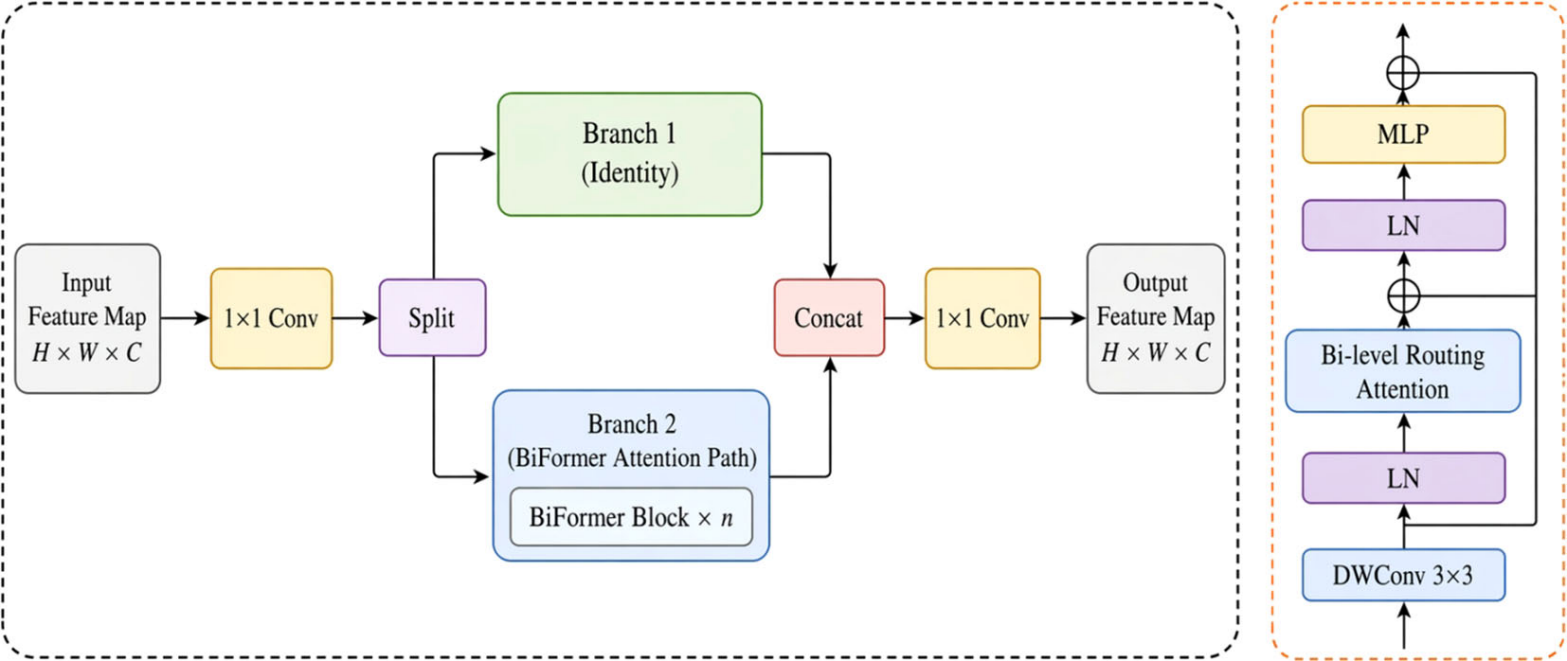
C2PSA_BIF architecture.

In the detection of apple, the multi-scale features extracted by the YOLO11 feature extraction network require further enhancement of local discriminative information. Traditional attention modules are prone to interference from foliage shadows and plastic film bagging, making it difficult to accurately focus on the core disease regions. To address this, this study designs the C2PSA_BIF module, as shown in Equation 2, which optimizes feature interaction through a bi-level routing attention mechanism to specifically enhance local key features of apple (Liu et al., 2021). Its structure is shown in the **Figure 5**, located after the SPPF module, undertaking global features and completing local refinement processing.

(2) $$Y^{\left(i\right)}=Softmax(\frac{QK^{⊤}}{\sqrt{d}})V+DWConv_{3\times 3}({\tilde{X}}^{\left(i\right)})$$

where ${\tilde{X}}^{\left(i\right)}$denotes the routed feature of the i-th window after window-level selection, Q, K, and V denote the query, key, and value matrices generated from the routed feature, d denotes the channel dimension of each attention head, DWConv3×3(·), denotes the depthwise convolution branch for position-sensitive enhancement, and Y(i) denotes the refined output of the i-th window.

Targeting the locally clustered yet globally scattered distribution characteristics of apples, the C2PSA_BIF module realizes dynamic screening of dapple phenotype via bi-level routing attention. First, the feature map is divided into 7×7 windows, and global features are extracted from each window. By calculating window correlation weights, the 4 most relevant neighboring windows are selected, and then key window features are aggregated to effectively suppress interference from irrelevant information such as branches, leaves, and fruit stems. For apple textures within windows, the module adopts a multi-head attention mechanism for refined processing: splitting into Q/K/V vectors, then scaling to strengthen the correlation of apple phenotype pixels; introducing 3×3 side-branch depthwise convolution to extract position-sensitive features, accurately capturing the edge morphology, and generating weights to amplify the response of apple phenotype.

With window-level feature screening and pixel-level attention enhancement as the core, the module combines the visual characteristics of apple. Through the combination of bi-level routing attention and PSABlock position-sensitive attention blocks, it achieves precise regulation of useful feature enhancement and useless feature suppression.

### 3.3 ASF2

In the task of apple counting in complex orchards, the neck of the YOLO11 model fails to fully explore the complementary value of multi-scale features. It neglects the correlation between large-scale features that carry the semantic information of apple population distribution and small-scale features that contain detailed information of individual small apples and occluded apples. This leads to redundant information or loss of key details in the fused features, thus compromising the counting accuracy of dense and small-sized apples. To address these issues, we improve the model by drawing on the architecture of ASF-YOLO and introducing the SSFF and TFE modules to make targeted remedies for the aforementioned defects.

**Figure 6:**
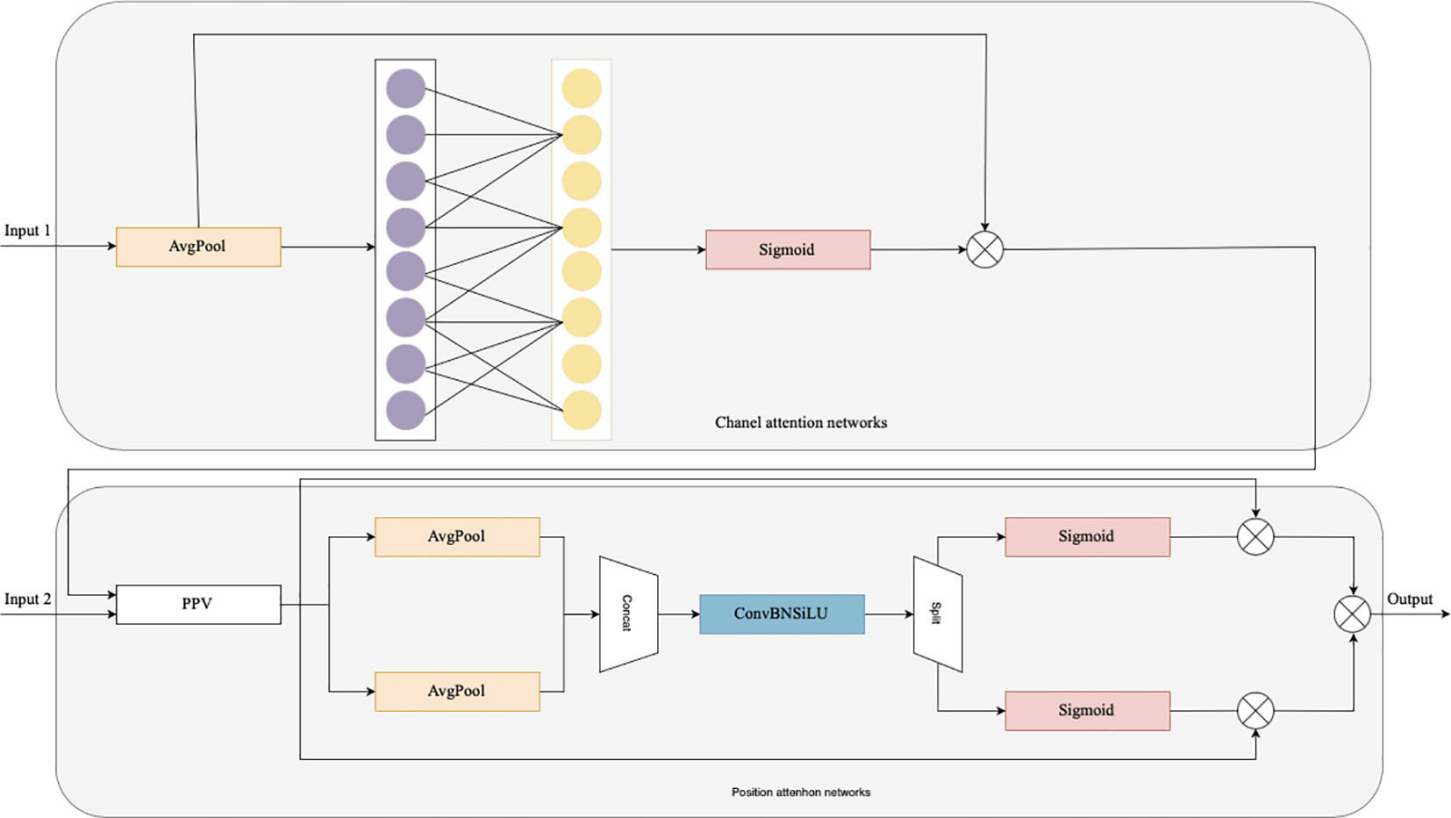
SSFF architecture.

SSFF is a lightweight parameter-free multi-scale feature fusion module with a concise and efficient structure, as shown in Equations 3-5, where Equation 4 describes the upsampling operation for the large-scale feature. Its core function is to achieve rapid alignment and aggregation of large, medium, and small scale apple features without introducing additional learnable parameters, thus avoiding an increase in model parameter count, and its structure is illustrated in the **Figure 6**. First, it receives three-scale spatial feature inputs and takes the size of the medium-scale feature map as the target alignment reference. Second, for large-scale features, it performs parallel computation of adaptive max-pooling and adaptive average-pooling followed by element-wise addition, which reduces the feature size while maximizing the retention of global semantic information about the apple population. For small-scale features, it adopts nearest-neighbor interpolation for upsampling, which enlarges the feature size while completely preserving detailed information of individual apples, tiny apples, and occluded apples. Finally, it concatenates the three aligned scale features along the channel dimension and outputs a feature map fused with multi-scale complementary information. The lightweight design ensures that counting accuracy is improved without sacrificing inference speed.

(3) $${\tilde{P}}_{3}=MaxPool_{S_{4}}(P_{3})+AvgPool_{S_{4}}(P_{3})$$

(4) $${\tilde{P}}_{5}=Up_{S_{4}}(P_{5})$$

(5) $$F_{SSFF}=Concat_{C}({\tilde{P}}_{3},P_{4},{\tilde{P}}_{5})$$

where $P_{3},\;P_{4}\;and\;P_{5}$ denote the small-, medium-, and large-scale feature maps, respectively, Up(·) denotes nearest-neighbor interpolation, and MaxPool(·) and AvgPool(·) denote adaptive max-pooling and adaptive average-pooling, respectively. F_SSFF_ denotes the fused output feature after scale alignment and channel-wise concatenation.

TFE is a deep cross-scale feature aggregation module based on 3D convolution, as shown in Equation 6, featuring a clearly layered structure. Its core purpose is to explore the internal correlation of apple features at different scales and enhance feature representation capability.

(6) $$Y=Squeeze(Conv_{3D}(Stack({\hat{P}}_{3},{\hat{P}}_{4},{\hat{P}}_{5})))$$

where ${\hat{P}}_{3},{\hat{P}}_{4},and{\hat{P}}_{5}$ denote the channel-unified and spatially aligned multi-scale features, Stack(·) denotes the operation of constructing an explicit scale dimension, Conv3D(·) denotes 3D convolution-based cross-scale interaction, Squeeze(·) denotes the removal of redundant dimensions, and Y denotes the final fused output feature.

**Figure 7:**
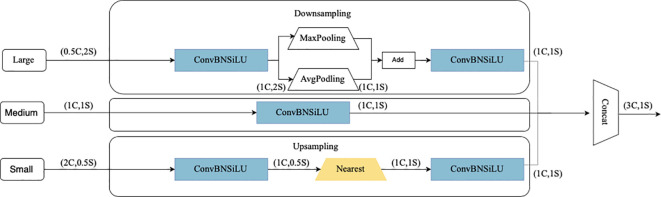
TFE architecture.

The module is composed of four core layers in total, and its structure is shown in the **Figure 7**. The feature channel unification layer consists of three independent 1×1 convolutions, which unify the input three-scale features with different channel numbers to the target channel number, eliminating channel differences to lay a foundation for subsequent feature fusion. The scale alignment layer upsamples the p4 and p5 features to the spatial size of the p3 features via nearest-neighbor interpolation, achieving spatial alignment of all input features. In the 3D feature interaction layer, a new dimension is first added to the aligned features to convert them into the 3D format; then the features are concatenated along this new dimension to form a feature tensor, which is subsequently processed by convolution, batch normalization, LeakyReLU activation, and max-pooling in sequence to realize deep cross-scale feature interaction and information screening. The feature compression layer removes redundant dimensions and converts the processed 3D feature tensor back to the 2D feature map format, outputting high-quality fused apple features to provide strong support for apple counting in complex orchards.

### 3.4 Experimental environment

The training hyperparameters are detailed in **Table 2**, with experimental configuration parameters summarized in **Table 3**.

**Table 2:** Training hyperparameters configuration.

Parameter name	Parameter value	Parameter name	Parameter value
epoch	300	batch	24
patience	30	workers	4
amp	False	optimizer	SGD
Image Input Resolution	640×640	lr0	0.01
size	640	lrf	0.01

**Table 3:** Experimental environment configuration.

Configuration name	Version & model
GPU Model & Quantity	NVIDIA GeForce RTX 4090 D (24 GB)
CPU Model	Intel Xeon(R) Platinum 8270
System RAM	128GB
Operating System	Windows 11 Home Chinese Edition (22H2 build)
Deep Learning Framework	PyTorch 2.0.1
CUDA Version	11.8.0
Programming Language	Python 3.8

## 4 Evaluation metrics

### 4.1 Traditional evaluation metrics

To evaluate the performance of the improved YOLO11 model, traditional metrics including precision (P), recall (R), and mean average precision (mAP) are employed. The calculation formulas for these metrics are presented in Equations 7-9 as follows, where Equation 8 defines the recall metric.

(7) $$P=\frac{TP}{TP+FP}\times 100\%$$

(8) $$R=\frac{TP}{TP+FN}\times 100\%$$

(9) $$mAP=\frac{1}{N}\sum_{k=1}^{N}AP(k)\times 100\%$$

In addition to detection metrics, two standard counting metrics, namely mean absolute error (MAE) and root mean square error (RMSE), were introduced to evaluate the counting accuracy of the model. Their Equations 10, 11 as follows:

(10) $$MAE=\frac{1}{N}\sum_{i=1}^{N}\left|{\hat{C}}_{i}-C_{i}\right|$$

(11) $$RMSE=\sqrt{\frac{1}{N}}\sum_{i=1}^{N}{\left({\hat{C}}_{i}-C_{i}\right)}^{2}$$

where *N* denotes the number of test images, ${\hat{C}}_{i}$ represents the predicted fruit count of the i-th image, and $\;C_{i}$ denotes the corresponding ground-truth count.

### 4.2 New evaluation metric for occlusion-aware recognition and counting

In natural orchard scenes, green apples are frequently affected by complex occlusion caused by interlaced branches, leaves, and mutual fruit overlap. In addition, the high similarity in color and texture between green apples and the surrounding foliage further increases the difficulty of accurate recognition. Under such conditions, fruit-counting models usually suffer from two critical error types, namely false detections and missed detections. False detections lead to over-counting, whereas missed detections cause under-counting. Therefore, both error types directly contribute to counting deviation and should be considered simultaneously when evaluating counting-oriented detection models.

To analyze the effect of occlusion in a more targeted manner, the annotated fruits were divided into four categories according to the proportion of occluded area, namely no occlusion, mild occlusion, moderate occlusion, and severe occlusion. The detailed classification criteria are listed in **Table 4**.

**Table 4:** Occlusion level categorization of green apples in natural scenes: A foundation for accurate detection and counting.

Occlusion level	Occluded area proportion	Typical scene description
No Occlusion		The fruit is fully visible with no coverage by branches or leaves.
Mild Occlusion		Part of the fruit is occluded by branches or leaves, but its main features remain clear.
Moderate Occlusion		Part of the fruit is occluded by branches or leaves, but its main features remain clear.
Severe Occlusion		The fruit is almost invisible, with only a small amount of its edges exposed.

To address this issue, this study constructed a task-oriented Overall Robustness and Counting Metric (ORCM) for occlusion-aware evaluation. Unlike the original formulation that only emphasized false detections, the revised ORCM jointly incorporates false-detection and missed-detection errors, thereby making the metric more consistent with the objective of fruit counting.

For the o-th occlusion level, the false-detection ratio and false-negative ratio are defined as follows, as shown in Equations 12 and 13:

(12) $$FDR_{o}=\frac{FP_{o}}{TP_{o}+FP_{o}}$$

(13) $$FNR_{o}=\frac{FN_{o}}{TP_{o}+FN_{o}}$$

where ${\text{TP}}_{\text{o}}$, ${\text{FP}}_{\text{o}}$, and $FN_{o}$ denote the numbers of true positives, false positives, and false negatives under the o-th occlusion level, respectively.

Since false detections and missed detections exert equivalent influence on counting deviation, an equal-weight error term was adopted in this study, as shown in Equation 14:

(14) $$E_{o}=\alpha \cdot FDR_{o}+(1-\alpha )\cdot FNR_{o},\;\alpha =0.5$$

Accordingly, the ORCM is defined as, as shown in Equation 15:

(15) $$ORCM=1-\frac{\sum_{o=0}^{3}\lambda_{o}\cdot E_{o}}{\sum_{o=0}^{3}\lambda_{o}}$$

where O∈{0,1,2,3} represents the four occlusion levels from no occlusion to severe occlusion, and $\lambda_{o}$ denotes the weight assigned to each occlusion level. In this study, the occlusion-level weights were fixed as $\lambda_{0}=1,\lambda_{1}=2,\lambda_{2}=3,\text{and}\lambda_{3}=4$,corresponding to no occlusion, mild occlusion, moderate occlusion, and severe occlusion, respectively.

Furthermore, the theoretical relationship between the revised ORCM and counting deviation can be expressed explicitly. For the o-th occlusion subset, the predicted count, the ground-truth count, and the corresponding counting deviation are defined as shown in Equations 16 and 17:

(16) $${\hat{C}}_{o}=TP_{o}+FP_{o},\;C_{o}=TP_{o}+FN_{o}$$

(17) $$CE_{o}=|{\hat{C}}_{o}-C_{o}|=|FP_{o}-FN_{o}|\leq FP_{o}+FN_{o}$$

Equation 17 indicates that both false detections and missed detections directly affect the final counting result. Therefore, compared with a metric based solely on false detections, the revised ORCM provides a more task-consistent evaluation of counting robustness under different occlusion levels. The value range of ORCM is 0 ≤ ORCM ≤ 1, and a higher ORCM indicates stronger robustness and better counting consistency in occluded orchard environments.

### 4.3 ACE robustness evaluation under illumination perturbation

In orchard environments, illumination variations may substantially alter the apparent color saturation, local contrast, and texture visibility of green apples, thereby affecting detection confidence and counting consistency. To quantitatively evaluate the robustness of the proposed YOLO11-SBS model under such conditions, an ACE metric was introduced to measure the average variation in model performance caused by controlled illumination perturbations. Rather than serving as a training objective, the ACE metric was used as a *post hoc* evaluation criterion to assess whether model predictions remained stable when illumination-dependent visual cues changed.

For an illumination perturbation level m, the ACE metric (Zhao et al., 2025) is defined in Equation 18:

(18) $$ACE=\frac{1}{n}\sum_{m=1}^{n}\left|E[Y(m)-Y(0)]\right|$$

Herein, n represents the total number of samples included in the experimental dataset. E(·) denotes the mathematical formulation for calculating the expected value, which serves as a fundamental statistical measure to aggregate and analyze data across multiple samples. Y(·) is employed to indicate the model evaluation metric specifically tailored for green apple counting accuracy, reflecting the model’s performance in terms of correctly identifying and enumerating the apples. Furthermore, m signifies different levels of lighting intensity perturbations introduced to simulate real-world orchard conditions, where lighting can vary significantly due to factors such as time of day, cloud cover, and shading from foliage. These perturbations are crucial for assessing the model’s robustness and generalizability under diverse environmental conditions. Y(0) represents the baseline counting result obtained under standard lighting conditions, serving as a reference point for comparison against results obtained under perturbed lighting scenarios.

## 5 Training results and analysis

### 5.1 Comparison of different detection algorithms

Currently, research in the field of object recognition is predominantly centered around end-to-end deep convolutional neural networks, particularly the YOLO series models known for their fast recognition speed and high accuracy. In this study, we selected YOLOv5, YOLOv6, YOLOv8, YOLOv9, YOLO11, the recently released YOLOv12 model, and YOLO11-SBS. Under the same dataset and experimental configuration conditions, we conducted model training and testing to determine the basic network architecture for the apple recognition model. The specific performance evaluation results are presented in **Table 5**.

**Table 5:** Comparison of test metrics for different models.

Model	P	R	mAP@50	mAP@50:95	Parameters	FPS
YOLOv5	88.0%	78.5%	89.5%	66.5%	2,181,859	164.5
YOLOv6	88.4%	78.4%	89.0%	65.8%	4,155,123	148.5
YOLOv8	85.6%	80.1%	88.8%	66.1%	2,684,563	149.2
YOLOv9	87.8%	79.0%	88.4%	65.8%	1,730,019	89.1
YOLOv10	86.8%	76.9%	87.7%	65.7%	2,694,806	116.7
YOLO11	85.5%	79.8%	88.9%	67.0%	2,582,347	130.4
YOLOv12	84.5%	79.4%	87.8%	65.0%	2,569,202	131.0
Rt-detr	84.9%	79.6%	83.4%	71.5%	19,987,336	209.8
YOLO11-SBS	86.7%	83.7%	92.0%	75.2%	2,569,755	128.8

As can be seen from **Table 3**, the proposed YOLO11-SBS network model in this paper achieves a detection precision of 86.7% and a recall of 83.7% for apple fruits. Although the improved YOLOv11 model shows a slight decrease in precision compared to YOLOv5, its significant improvement in recall endows it with remarkable advantages in complex orchard environments. It can more comprehensively detect apple fruits amidst occlusions and cluttered backgrounds.

The mAP@50 is 92.0%. This metric is an important evaluation criterion in the field of object detection. It measures the average precision of the model in detecting targets under different Intersection-over-Union (IoU) thresholds (in this case, 0.5). A high mAP@50 value indicates that the model can accurately identify targets under relatively lenient detection criteria. The mAP@50:95 is 75.2%. This metric further takes into account the model’s average precision across the IoU range from 0.5 to 0.95, reflecting the model’s comprehensive performance under different levels of detection strictness. The excellent performance of the improved YOLOv11 in this metric fully demonstrates its stability and reliability in detecting apple fruits in complex scenarios.

**Figure 8:**
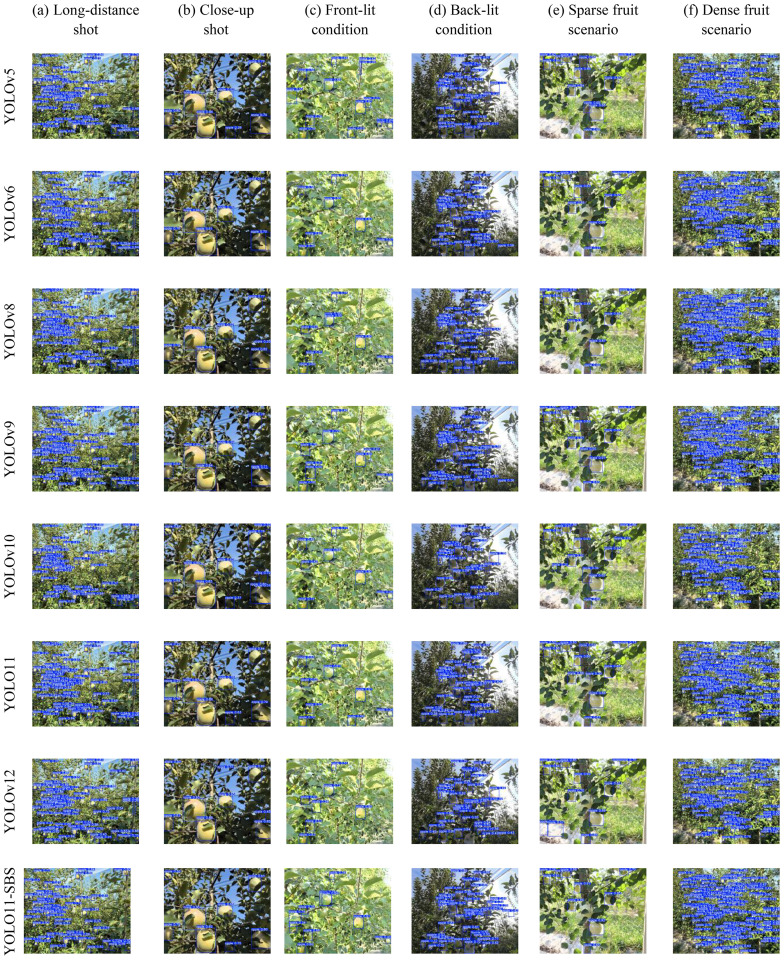
Different scenario-based model performance analysis: **(a)** long-distance shot; **(b)** close-up shot; **(c)** front-lit condition; **(d)** back-lit condition; **(e)** sparse fruit scenario; and **(f)** dense fruit scenario.

The visual comparison in **Figure 8** vividly demonstrates the outstanding performance of YOLO11-SBS compared to other models in the green apple detection task across multiple scenarios.

In the “long-distance” (a) scenario, models such as YOLOv5-v9 and YOLOv10-v12 exhibit varying degrees of missed detections, with some apples completely undetected. In contrast, YOLO11-SBS successfully identifies a significantly larger number of apples. This indicates that even when apples are far away, with relatively small and indistinct visual features, YOLO11-SBS has enhanced its ability to capture target objects.

Under “close-distance” (b) and “front-lit” (c) conditions, although most models perform reasonably well, YOLO11-SBS still demonstrates a more comprehensive detection coverage. It can accurately locate apples that may be partially occluded or have subtle visual differences from the background. In the highly challenging “back-lit” (d) scenario, the contrast between apples and the background is significantly reduced. Other models suffer from high missed detection and false detection rates (as indicated by blue markers). However, YOLO11-SBS shows remarkable robustness, maintaining relatively high detection accuracy and completeness. This is attributed to its advanced feature extraction and fusion mechanisms, which enable it to better adapt to low-contrast environments.

When it comes to “few-apples” (e) and “many-apples” (f) scenarios, the advantages of YOLO11-SBS become even more prominent. In the “few-apples” case, some models are prone to false detections, misidentifying non-apple objects as apples, while YOLO11-SBS effectively avoids such errors, demonstrating high precision. In the “many-apples” scenario, where apples are densely distributed, the probability of occlusion and overlap is high. The performance of other models significantly declines, with a large number of missed detections.

YOLO11-SBS performs exceptionally well in this complex situation. Its multi-scale feature processing capability and improved attention mechanism allow it to accurately distinguish individual apples even when they are crowded, while ensuring high recall and high precision. Overall, through a comprehensive comparison across different scenarios, YOLO11-SBS stands out as a more reliable and better-performing model in green apple detection, capable of adapting to various real-world orchard environments with excellent stability and accuracy.

### 5.2 Ablation experiment result analysis

Ablation experiments were conducted on the same apple dataset under the same experimental environment for the improved YOLOv8 apple recognition model. Through model training, the parameters of the improved and optimized models for the backbone network, Neck layer, and Head layer in the YOLOv8 baseline network were obtained. These models were then tested and analyzed on a unified test set, and the performance evaluation results are shown in **Table 6**.

**Table 6:** Ablation experiment results of the improved YOLO11 apple recognition model.

BIFORMER	ASF2	SAConv	P	R	mAP@50	mAP@50:95	Parameters	FPS
✓	×	×	87.2%	79.2%	89.5%	67.2%	3,096,772	76.9
×	✓	×	88.3%	77.4%	89.2%	66.8%	3,079,364	87.7
×	×	✓	86.6%	78.9%	89.0%	66.2%	2,705,161	104.5
✓	✓	×	87.7%	80.3%	90.0%	67.6%	2,973,958	87.2
✓	×	✓	84.9%	81.9%	90.7%	71.2%	2,956,550	77.0
×	✓	✓	88.4%	80.8%	91.1%	70.5%	2,722,569	106.1
✓	✓	✓	86.7%	83.7%	92.0%	75.2%	2,569,755	128.8

Note: “√” indicates that the module is incorporated, while “×” means it is not used.

As can be seen from **Table 6**, when only BiFormer is integrated, the model achieves a P of 87.2%, a R of 79.2%, with mAP@50 and mAP@50:95 reaching 89.5% and 67.2% respectively. This configuration demonstrates the effectiveness of BiFormer in enhancing the overall detection performance of the model. When only ASF2 is introduced, the precision rises to 88.3%, but the recall slightly drops to 77.4%, indicating that ASF2 has the potential to optimize the model’s ability to recognize target objects, yet may somewhat affect its ability to capture some targets. When only SAConv is adopted, the precision and recall are 86.6% and 78.9% respectively, showing an intermediate effect in balancing the two.

When BiFormer and ASF2 are combined simultaneously, the model maintains a relatively high precision while significantly improving the recall to 80.3%. The mAP@50 and mAP@50:95 also increase accordingly, reflecting the synergistic effect of the two in enhancing the model’s detection performance. It is particularly noteworthy that when BiFormer is combined with SAConv, although the precision decreases to 84.9%, the recall substantially increases to 81.9%, and the mAP metrics are significantly optimized, indicating that this combination is highly effective in enhancing the model’s ability to capture target objects. Similarly, the combination of ASF2 and SAConv also brings about a dual improvement in recall and mAP.

The most striking result is when BiFormer, ASF2, and SAConv are all integrated into the model simultaneously. The model achieves the highest recall of 83.7%, while maintaining a precision of 86.7%. The mAP@50 and mAP@50:95 reach excellent levels of 92.0% and 75.2% respectively. This result not only proves the individual contributions of each component but also highlights their synergistic enhancement effect, enabling the model to perform exceptionally well in the apple detection task in complex orchard environments. It can effectively cope with challenges such as occlusion and cluttered backgrounds, achieving high-precision and high-recall detection results.

### 5.3 Heatmap comparison experiment

**Figure 9:**
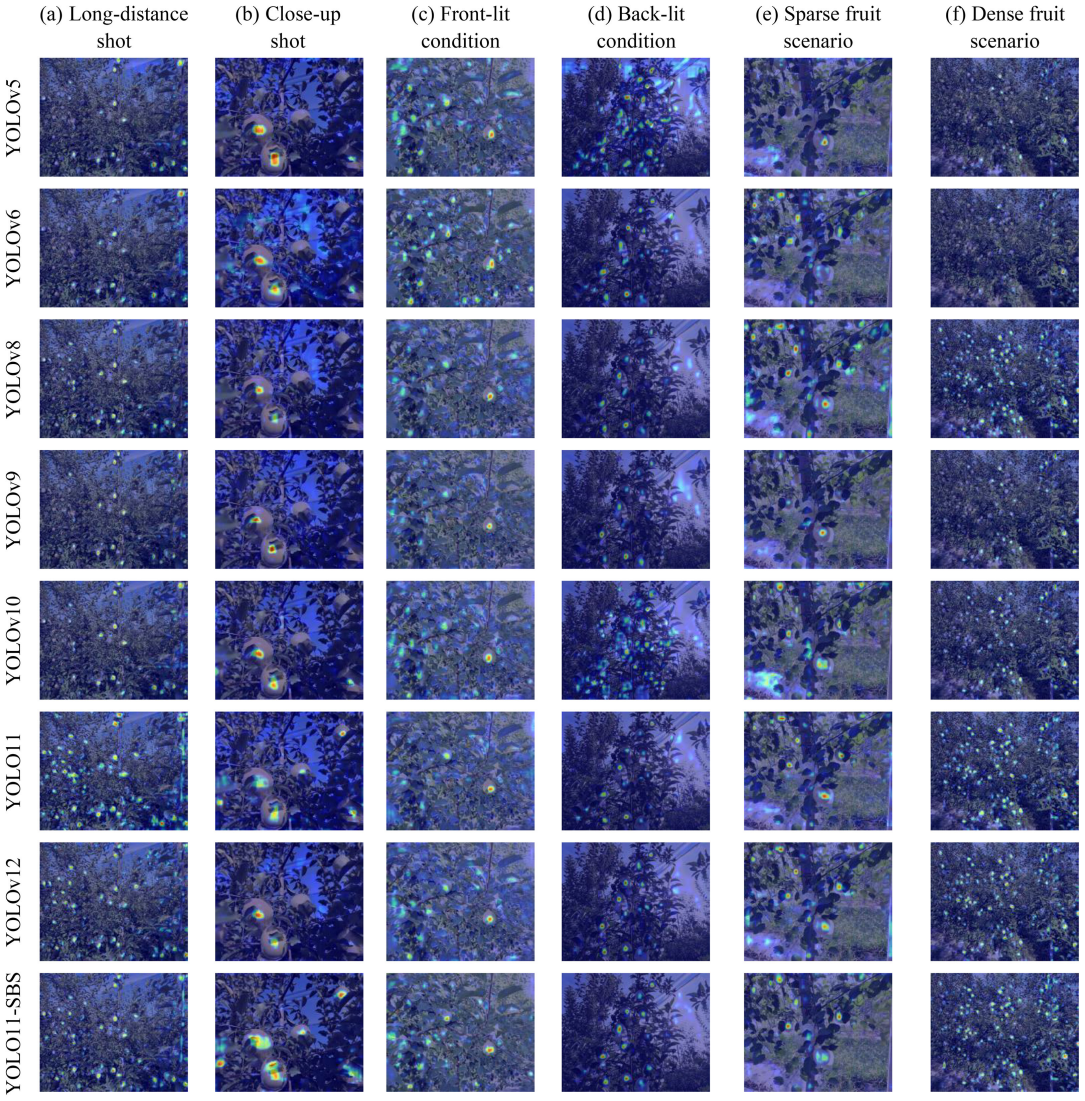
Comparison of heatmaps among different models under different orchard scenarios: **(a)** long-distance shot; **(b)** close-up shot; **(c)** front-lit condition; **(d)** back-lit condition; **(e)** sparse fruit scenario; and **(f)** dense fruit scenario.

The heatmaps presented in the **Figure 9** profoundly reveal the superiority of YOLO11-SBS over other YOLO-series models in the context of green apple detection. In the “long-distance” (a) scenario, the heatmaps of models like YOLOv5-v10 show relatively dispersed and less-focused high-activation regions, indicating difficulties in precisely locating distant apples. In contrast, YOLO11-SBS displays more concentrated and well-defined high-activation regions that closely align with the actual positions of the apples, demonstrating its enhanced ability to accurately locate distant targets.

Under “close-distance” (b) and “front-lit” (c) conditions, although other models also show some high-activation regions around the apples, the heatmap of YOLO11-SBS exhibits more complete coverage of the apple regions. This means it can capture a broader range of features related to the apples, including edge and partially occluded features, which are crucial for accurate detection. In the challenging “back-lit” (d) scenario, where apples are difficult to distinguish from the background, the heatmaps of other models become highly fragmented and noisy, with many false high-activation regions in non-apple areas. However, YOLO11-SBS maintains relatively clear and focused high-activation regions on the apples, demonstrating its robustness in low-contrast and complex lighting conditions.

When considering “few-apples” (e) and “many-apples” (f) scenarios, the advantages of YOLO11-SBS become even more evident. In the “few-apples” case, the heatmaps of some models show high-activation regions in non-apple areas, indicating a high probability of false detections. On the other hand, the high-activation regions of YOLO11-SBS are precisely concentrated on the few existing apples, indicating high detection precision.

In the “many-apples” scenario, where apples are densely arranged, the heatmaps of other models show a chaotic pattern with overlapping and unclear high-activation regions, leading to high missed detection and misclassification rates. In contrast, the heatmap of YOLO11-SBS displays clearly separated high-activation regions for individual apples even in such a crowded environment. This demonstrates its excellent multi-scale feature processing and target discrimination capabilities. Overall, through the analysis of these heatmaps in different scenarios, it is clear that YOLO11-SBS outperforms other models in terms of accuracy, completeness, and robustness of target detection, making it a highly competitive model for green apple detection in real-world orchard environments at the level required by top-tier journals.

### 5.4 10-fold cross-validation

In the task of green apple detection in complex orchards, to ensure the reliability and comprehensiveness of model performance evaluation, we adopted a rigorous 10-fold cross-validation strategy. Specifically, the entire carefully collected and annotated dataset of green apples in orchards was randomly and evenly divided into 10 mutually exclusive subsets. Each subset was designed to maintain, as much as possible, a data distribution consistent with that of the original dataset, so as to minimize the impact of data partitioning bias on the model evaluation results.

In each round of cross-validation, 9 of these subsets were selected as the training set for model parameter learning and optimization, while the remaining 1 subset served as the validation set for independently assessing the model’s performance after that round of training. This process was repeated 10 times, ensuring that each subset had the opportunity to act as the validation set exactly once, as shown in **Table 7**. Through this systematic and comprehensive validation approach, we were able to evaluate the model from multiple perspectives and under different data combinations. This effectively avoided the accidental errors caused by a single data partitioning, thereby obtaining more stable and reliable model performance metrics.

**Table 7:** 10-fold cross-validation.

Fold	P	R	mAP@50	mAP@50:95
1	85.3%	82.1%	90.5%	74.0%
2	87.2%	84.5%	92.8%	76.3%
3	86.1%	83.2%	91.7%	75.0%
4	88.0%	85.0%	93.5%	77.2%
5	85.8%	82.8%	91.2%	74.7%
6	87.5%	84.8%	93.0%	76.8%
7	86.4%	83.5%	91.9%	75.4%
8	88.3%	85.3%	93.8%	77.7%
9	85.6%	82.5%	90.9%	74.3%
10	87.8%	85.1%	93.3%	74.5%
Average	86.7%	83.7%	92.0%	75.2%
Std	1.1%	1.2%	1.3%	0.7%

### 5.5 Validation with ORCM

As shown in **Table 8**, the models exhibited markedly different performance patterns under different occlusion levels. Compared with conventional metrics, the ORCM used in this study provides a more counting-oriented evaluation because it jointly considers false detections and missed detections under no, mild, moderate, and severe occlusion conditions.

**Table 8:** Comparison of error rates in green apple recognition and ORCM metrics among different models under various occlusion levels.

Model	No Occlusion	Mild Occlusion	Moderate Occlusion	Severe Occlusion	ORCM	MAE	RMSE
YOLOv5	20.6%	10.7%	16.7%	7.6%	0.746	7.83	9.62
YOLOv6	23.1%	13.8%	19.8%	8.2%	0.721	7.21	8.95
YOLOv8	24.7%	11.9%	20.8%	10.4%	0.802	5.97	7.58
YOLOv9	31.6%	18.6%	21.3%	8.4%	0.781	6.34	8.02
YOLOv10	14.3%	7.4%	13.2%	6.6%	0.824	5.42	7.01
YOLO11	25.6%	14.2%	9.5%	11.4%	0.846	4.98	6.52
Rt-detr	16.8%	8.1%	10.2%	6.1%	0.861	4.62	6.08
YOLO11-SBS	14.6%	6.7%	8.3%	5.2%	0.878	3.86	5.34

In practical fruit counting, false detections lead to over-counting, whereas missed detections result in under-counting. Therefore, evaluating only one type of error cannot fully reflect the actual counting quality of a model. By integrating both error sources and assigning larger weights to more severe occlusion levels, ORCM offers a more targeted assessment of counting robustness in natural orchard environments.

Among all the compared models, YOLO11-SBS achieved the highest ORCM value, indicating that it maintained the best balance between suppressing false detections and reducing missed detections across different occlusion levels. This result further demonstrates the superiority of the proposed model in occlusion-aware recognition and counting tasks.

The ORCM metric played a pivotal role in this evaluation because it provided a more comprehensive and targeted assessment of model performance in the presence of occlusions. When applied to green apple counting models in real-world scenarios with occlusions, traditional evaluation metrics such as the F1 score and mAP exhibit significant limitations.

The F1 score solely focuses on global precision and recall, failing to distinguish the impact of false positives and false negatives on actual yield statistics under different occlusion levels. Similarly, mAP relies on IoU-based matching, and occlusions may distort the overlap relationship between predicted boxes and ground-truth annotations, thereby limiting its ability to directly reflect counting reliability in complex orchard scenes.

**Figure 10:**
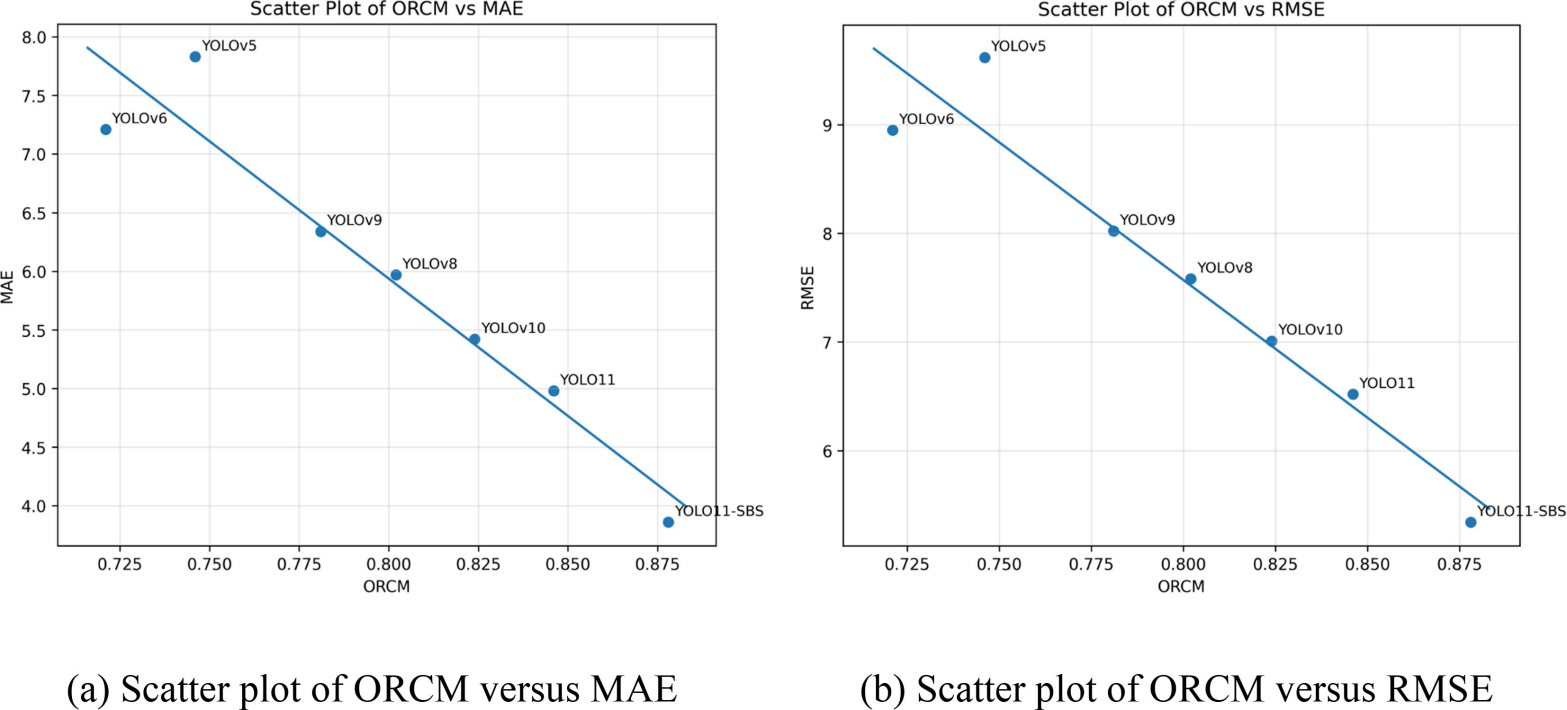
Correlation analysis between ORCM and standard counting metrics: **(a)** ORCM versus MAE; **(b)** ORCM versus RMSE.

As shown in the scatter plots in **Figure 10**, ORCM exhibited a strong negative correlation with MAE (Pearson r=−0.962, Spearman ρ=−0.964, p<0.001) and RMSE (Pearson r=−0.962, Spearman ρ=−0.964, p<0.001). These results indicate that models with higher ORCM values tend to yield lower counting errors. In particular, YOLO11-SBS is located closest to the lower-right region of both scatter plots, indicating that it achieves the highest ORCM together with the lowest MAE and RMSE among all compared models.

On the other hand, ORCM takes into account the error rates under different occlusion categories and assigns a higher weight to severe occlusion cases. This approach effectively highlights the model’s performance in dealing with complex occlusion scenarios, which is of utmost importance for practical green apple counting applications.

Compared with other state-of-the-art models, the superiority of our YOLO11-SBS model in terms of ORCM, together with its strong consistency with MAE and RMSE, clearly demonstrates its advantage in accurately recognizing and counting green apples under diverse and challenging occlusion conditions in orchards, making it a more reliable choice for real-world deployment.

### 5.6 Verification of causality between light intensity and detection outcomes

A smaller ACE value indicates that model predictions are less sensitive to illumination perturbations. As shown in **Table 9**, YOLO11-SBS achieves the lowest or joint-lowest values in most evaluation indicators under simulated lighting conditions. Specifically, its precision is 4.5%, which is tied for the lowest value among the compared models; its recall is 4.6%, lower than that of the baseline YOLO11 and all other YOLO-series models except none; its mAP@50 is 3.1%, which is also the joint-lowest value; and its mAP@50:95 reaches 5.0%, which is the lowest among all compared methods. These results indicate that the prediction results of YOLO11-SBS are least affected by illumination perturbation, and its performance degradation remains the smallest under intervention.

**Table 9:** Performance variation under simulated lighting conditions.

Model	ΔP	ΔR	ΔmAP@50	ΔmAP@50:95	ACE
YOLOv5	6.8%	7.9%	4.7%	7.1%	6.60%
YOLOv6	10.6%	4.2%	4.8%	8.3%	7.00%
YOLOv8	4.2%	5.4%	3.8%	5.9%	4.80%
YOLOv9	9.1%	6.5%	3.6%	7.3%	6.60%
YOLOv10	7.5%	5.0%	4.0%	7.3%	6.00%
YOLO11	4.5%	6.2%	3.1%	7.2%	5.30%
Rt-detr	5.2%	6.4%	3.5%	7.3%	5.60%
YOLO11-SBS	4.5%	4.6%	3.1%	5.0%	4.30%

Overall, YOLO11-SBS achieves the best ACE-related performance among the compared models. In other words, the proposed model shows the smallest causal effect of lighting intervention on detection outcomes, indicating that it depends less on superficial illumination-sensitive cues and more on relatively stable structural and semantic characteristics of apple targets. This finding further confirms that YOLO11-SBS possesses stronger illumination robustness and better generalization ability in complex orchard environments, which is of substantial practical significance for reliable green apple detection and counting under real-world field conditions.

## 6 Conclusion

In complex orchard environments, precise apple counting is of substantial importance for orchard management, yield estimation, and automated harvesting. However, conventional YOLO-based models still exhibit several inherent limitations, including fixed convolutional weights, insufficient feature screening capability, and strong susceptibility to interference from branch and leaf shadows, fruit bagging, and other background noise. In addition, their limited ability to exploit complementary multi-scale information often leads to the loss of fine-grained details, thereby reducing counting accuracy for small, dense, and partially occluded apples.

To address these issues, this study proposed the YOLO11-SBS model for robust and accurate green apple detection and counting in complex orchards. Specifically, the C3k2_SAC module was introduced to enhance adaptive feature extraction through dynamic convolutional weight calibration, while the C2PSA_BIF module improved local feature interaction and discriminative representation. Meanwhile, the SSFF and TFE modules strengthened multi-scale feature alignment and cross-scale feature fusion, enabling the model to better distinguish apples from cluttered backgrounds and to handle dense and occluded fruit targets more effectively.

To provide a more task-consistent evaluation of model performance, this study further incorporated the ACE metric to assess robustness under illumination perturbation and constructed an ORCM to quantify robustness and counting-related error characteristics under different occlusion levels. Experimental results demonstrated that YOLO11-SBS achieved clear advantages over the baseline YOLO models by effectively reducing missed detections and false detections while maintaining high precision and recall, particularly in cases where green apples exhibited strong visual similarity to the surrounding foliage. These findings indicate that the proposed model provides an effective and reliable solution for green apple counting in complex agricultural environments and has promising practical value for intelligent orchard management and automated harvesting applications.

At the same time, several limitations of the present study should be noted. First, the dataset was collected from a relatively limited geographical region and mainly focused on a single apple cultivar within a specific acquisition period, which may restrict the generalizability of the proposed model to other orchard environments, cultivars, growth stages, and seasonal conditions. Second, although YOLO11-SBS demonstrated strong robustness under natural illumination variation and occlusion, its performance under more extreme conditions, such as severe motion blur, adverse weather, nighttime environments, and cross-device domain shifts, still requires further validation. Third, the incorporation of multiple enhancement modules may increase model complexity to a certain extent, which could impose additional constraints on deployment in highly resource-limited edge devices.

## Data Availability

The raw data supporting the conclusions of this article will be made available by the authors, without undue reservation.
